# Anthropometric and Body Composition Correlates of Hypertension in Children and Adolescents with Intellectual Disabilities

**DOI:** 10.3390/jcm15031058

**Published:** 2026-01-28

**Authors:** Justyna Wyszyńska, Katarzyna Dereń, Artur Mazur, Piotr Matłosz

**Affiliations:** 1 Faculty of Medical Sciences and Psychology, Medical College of Rzeszow University, 35-959 Rzeszow, Poland; kderen@ur.edu.pl; 2 Medical Faculty, Medical College of Rzeszow University, 35-959 Rzeszow, Poland; armazur@ur.edu.pl; 3 Faculty of Physical Culture Sciences, Medical College of Rzeszow University, 35-959 Rzeszow, Poland; pmatlosz@ur.edu.pl

**Keywords:** intellectual disability, hypertension, isolated systolic hypertension, anthropometry, body composition, obesity, children, adolescents

## Abstract

**Background/Objectives**: Children and adolescents with intellectual disabilities (ID) have an elevated burden of obesity and cardiometabolic risk, yet factors associated with high blood pressure (BP) in this group remain insufficiently described. This study assessed the prevalence of hypertension (HTN) and isolated systolic hypertension (ISH) at a single visit and examined anthropometric and body composition correlates of elevated BP in children with ID. **Methods**: A cross-sectional study was conducted among 461 children and adolescents with ID aged 7–18 y attending special education schools in southeastern Poland. Anthropometric indicators (BMI, waist circumference [WC], hip circumference [HC], and waist-to-height ratio [WHtR]) and body composition parameters (BF%, MM%, FFM%, TBW%) were measured using standardized procedures. BP was assessed three times during one visit, and the average of the second and third readings was used. Receiver operating characteristic (ROC) analyses were used for exploratory assessment of discriminatory performance of anthropometric and body composition parameters, and multivariable logistic regression examined associations with elevated BP (HTN + ISH). **Results**: Overall, 13.9% of participants had HTN and 10.4% had ISH (combined prevalence: 24.3%). Abdominal obesity was present in 39.5% of participants, and elevated HC in 28.2%, both more common in girls. Higher BP categories were associated with greater WC, HC, BMI, and BF%, and lower MM%, FFM%, and TBW% (*p* < 0.0001). HC showed the highest discriminatory accuracy for HTN + ISH (AUC = 0.844), followed by MM%, BF%, and FFM%, whereas WHtR demonstrated limited discriminatory performance in ROC analyses. In multivariable models, WHtR ≥ 0.5 was associated with increased odds of elevated BP (OR = 4.25), whereas higher TBW% (≥55.38%) was inversely associated with elevated BP (OR = 0.17) in the total sample; similar patterns were observed in sex- and age-stratified analyses. **Conclusions**: Children with ID show a high prevalence of elevated BP at a single visit, including HTN-range and ISH-range values. Anthropometric indicators, particularly HC and WHtR, and BIA-derived body composition parameters reflecting higher fat mass and lower lean tissue proportion were associated with elevated BP. These exploratory findings suggest that simple anthropometric and body composition measures may help identify individuals who warrant further BP assessment, although longitudinal studies with repeated measurements are required before clinical application.

## 1. Introduction

Hypertension (HTN) is increasingly recognized as a major public health problem among children and adolescents, with its prevalence rising in parallel with the global obesity epidemic. Recent epidemiological data indicate that elevated blood pressure (BP) and HTN are increasingly prevalent in pediatric populations worldwide. A recent systematic review and meta-analysis including over 179,000 children and adolescents from 18 European countries estimated the pooled prevalence of HTN at approximately 4%, with substantial between-country variability and higher rates observed in adolescents and children with excess adiposity. Globally, similar trends have been reported, highlighting pediatric HTN as an emerging public health concern rather than a rare clinical finding [1].

Current clinical practice guidelines emphasize age-, sex-, and height-specific BP percentiles for the classification of BP status in children and adolescents. According to the 2016 European Society of Hypertension guidelines, HTN in children younger than 16 years is defined as systolic (SBP) and/or diastolic (DBP) at or above the 95th percentile, while fixed adult cut-off values are applied from the age of 16 years onward. These recommendations underscore the importance of accurate BP assessment and early identification of elevated BP during childhood to reduce long-term cardiovascular risk [2].

Elevated BP in childhood has become more common, whereas it was once considered only an adult issue. It is now recognized as an early marker of cardiovascular risk later in life. Children with intellectual disabilities (ID) represent a particularly vulnerable population as they exhibit higher rates of obesity, metabolic disturbances, and cardiovascular complications than their typically developing peers [3,4,5]. The prevalence of HTN in this group has been reported to reach up to 30% in some studies [4,6], indicating a substantially greater burden compared with the general pediatric population.

Several biological and behavioral factors contribute to the increased risk of HTN in children with ID, including excess body fat, low levels of physical activity, sedentary lifestyle, and dietary imbalances. Socioeconomic disadvantage and reduced access to preventive healthcare further exacerbate this problem [3,4,5]. Early detection is critical, as elevated BP during childhood tends to persist into adulthood, leading to long-term cardiovascular complications. However, despite the high risk, children with ID are rarely included in screening or prevention programs, and data on determinants of HTN in this population remain scarce. These challenges underscore the need for simple, feasible, and non-invasive screening approaches that can be implemented in school and community settings to facilitate early identification of elevated blood pressure in children with ID.

Children and adolescents with ID tend to exhibit unfavorable body composition profiles, characterized by higher fat mass and lower levels of muscle mass compared with typically developing peers. Previous studies have consistently reported a high prevalence of overweight and obesity in this population, alongside an increased burden of cardiometabolic risk factors, including elevated BP. Importantly, beyond overall adiposity, emerging evidence suggests that body composition components such as fat distribution and reduced lean mass may be associated with BP levels in children with ID, although data remain limited and heterogeneous. These findings highlight the need for further research using combined anthropometric and body composition assessments to better characterize blood pressure-related risk in this vulnerable population [7].

Several pathophysiological mechanisms may underlie the association between body composition and BP in children. Excess adiposity, particularly central fat accumulation, has been linked to increased sympathetic activity, insulin resistance, inflammation, and endothelial dysfunction, whereas lower muscle mass may be associated with impaired metabolic and vascular regulation. Pediatric evidence suggests that muscle mass is inversely associated with BP, independent of overall adiposity, supporting the assessment of both fat- and lean-mass–related parameters when evaluating BP risk in children [8,9].

Although waist circumference (WC) is the most commonly used anthropometric marker of central adiposity, hip circumference (HC) may provide complementary information on body fat distribution and gluteofemoral tissue mass, which have been linked to cardiometabolic risk profiles. In pediatric populations, HC has been shown to be associated with BP independently of WC and body mass index (BMI), potentially reflecting differences in peripheral fat storage and lean tissue distribution. Emerging evidence suggests that HC may capture aspects of somatic growth and body composition not fully represented by waist-based measures alone, supporting its consideration as an additional, simple anthropometric indicator in blood pressure risk assessment [7,10].

Anthropometric indices, particularly WC and HC, have been recognized as simple and reliable predictors of HTN in children, often outperforming BMI in identifying cardiometabolic risk [7,11,12]. In this context, combining waist- and hip-based measures may offer a more comprehensive characterization of body fat distribution relevant to BP regulation. In addition, bioelectrical impedance analysis (BIA) allows for non-invasive assessment of body composition, including fat and fat-free mass distribution, which provides a deeper understanding of the mechanisms linking excess adiposity with elevated BP. While BIA has been used in children with ID, its application for discriminating elevated BP categories in this group remains largely unexplored.

Although BMI remains the most widely used indicator of nutritional status in pediatric populations, it does not distinguish between fat mass and lean mass, nor does it capture differences in body fat distribution. As a result, BMI may inadequately reflect cardiometabolic risk, particularly in populations with altered growth patterns or reduced muscle mass, such as children with ID. In this context, body composition assessment provides additional, relevant information by enabling a more nuanced interpretation of the relationships between adiposity, lean tissue, and BP [13,14].

Therefore, the aim of this study was to examine the prevalence of HTN assessed at a single visit and to explore associations between anthropometric indices and body composition parameters and elevated BP in children and adolescents with ID. In particular, we sought to compare the discriminatory performance of WC, HC, and BIA-derived body composition measures in relation to elevated BP. ROC analyses and derived cut-off values were applied for exploratory and comparative purposes only and should not be interpreted as validated screening or diagnostic thresholds. The findings are intended to provide hypothesis-generating insights that require confirmation in longitudinal studies with repeated BP measurements. Methodologically, this study provides one of the first large-scale, standardized evaluations combining HC, WC, and BIA-derived body composition parameters in a pediatric population with ID.

## 2. Materials and Methods

### 2.1. Study Design and Study Sample

**Figure 1:**
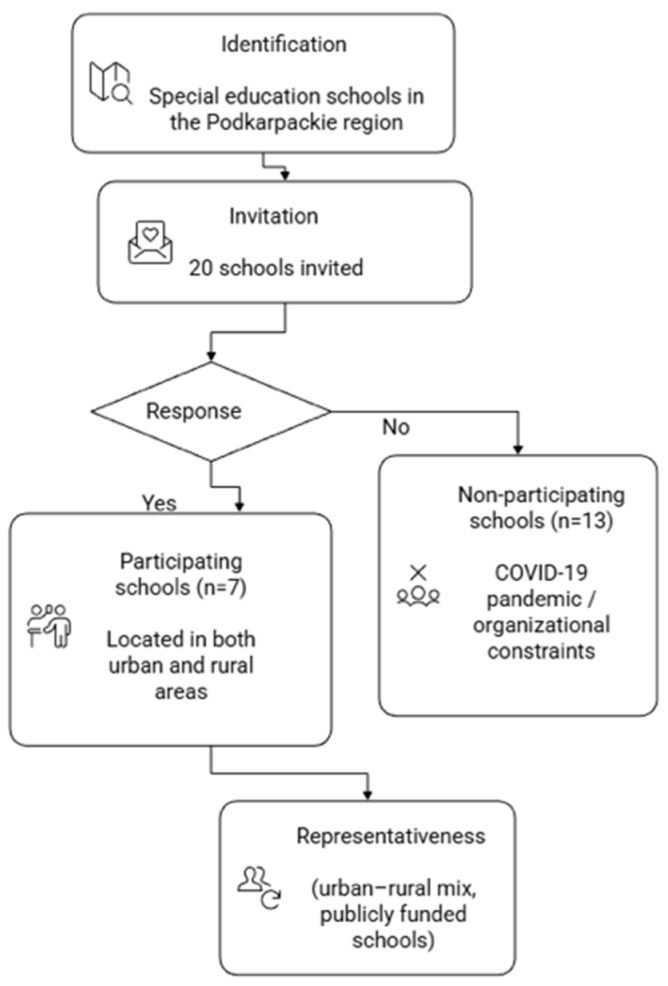
Flow diagram of school recruitment and participation.

This cross-sectional study was conducted in the Podkarpackie region of southeastern Poland during the 2019/2020 school year. Invitations to participate in this study were sent to all special education schools in the region (n = 20), and seven schools agreed to take part in the study. Although all special education schools in the region were invited, only seven agreed to participate, as recruitment took place during the COVID-19 pandemic. These schools were publicly funded institutions located in both urban and rural areas, which may be considered a proxy for socioeconomic representativeness. A flow diagram illustrating school recruitment and participation is provided in Figure 1.

Following approval from school directors, invitations and detailed information about the study were distributed to the parents or legal guardians of all pupils attending these schools. A total of 501 written consents were obtained.

**Figure 2:**
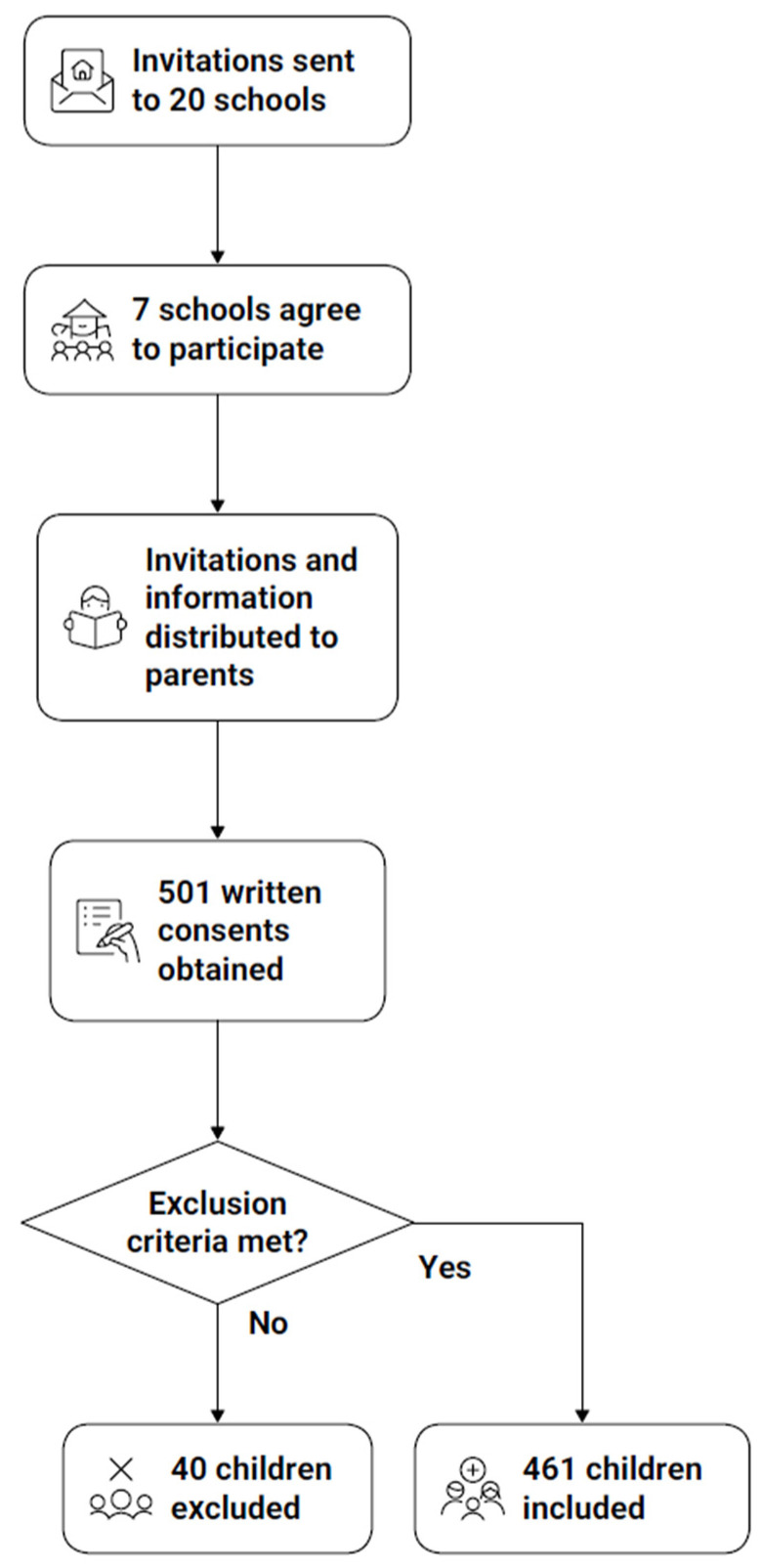
Recruitment and selection process of study participants.

Of these, 40 children were not examined for the following reasons: failure to meet the inclusion criteria (n = 5), absence on the day of the study (n = 25), or refusal or strong anxiety during the examination (n = 10). The final study sample therefore consisted of 461 children and adolescents with intellectual disabilities, aged 7–18 y. The recruitment and selection process is illustrated in Figure 2.

Inclusion criteria were children and adolescents aged 7–18 y enrolled in special education schools in the Podkarpackie region, whose parents or legal guardians provided written informed consent. All participants had a documented diagnosis of mild, moderate, severe, or profound intellectual disability established by a child psychiatrist or clinical psychologist and verified through school records. Participants were also required to follow simple verbal instructions during the measurement procedures, with assistance from caregivers or teachers when necessary.

Exclusion criteria included the presence of implantable electronic or metallic devices, wheelchair dependence or inability to stand barefoot on the BIA platform, pregnancy, and acute illness on the day of examination. Children with medical conditions affecting BP or hydration status, such as chronic kidney disease, congenital heart disease, diabetes, or endocrine disorders, were also excluded.

### 2.2. Anthropometric Measurements

Height was measured to the nearest 0.1 cm (Tanita HR-200 stadiometer, Tanita Corporation, Tokyo, Japan), with participants standing barefoot, heels together, and head in the Frankfort horizontal plane.

Body weight was measured to the nearest 0.1 kg using a Tanita BC-420MA body composition analyzer (Tanita Corporation, Tokyo, Japan), with participants wearing light indoor clothing and no footwear. Body mass index (BMI) was computed as weight (kg) divided by height squared (m^2^), and BMI percentiles were assigned based on Polish reference growth charts for children and adolescents [15]. Based on BMI percentile thresholds, participants were classified as underweight (below the 5th percentile), normal weight (5th–84th percentile), overweight (85th–94th percentile), or with obesity (95th percentile or higher) [16].

WC was assessed to the nearest 0.1 cm using a flexible, non-stretchable steel anthropometric tape (Lufkin W606PM, Cooper Industries, Mount Vernon, OH, USA). Measurements were obtained directly on the skin with participants standing upright, abdomen relaxed, arms at the sides, and feet together. The tape was placed horizontally at the midpoint between the lower border of the last palpable rib and the iliac crest. Each measurement was taken at the end of a gentle exhalation, ensuring no pressure was applied to the skin. Abdominal obesity was determined according to Polish age- and sex-specific WC percentile charts [17]. WC values below the 90th percentile were considered normal, whereas measurements at or above the 90th percentile were classified as indicating abdominal obesity [18].

HC was measured to the nearest 0.1 cm using the same non-elastic steel anthropometric tape. Measurements were taken with participants standing upright, feet together, and arms relaxed at the sides. The tape was positioned horizontally around the maximum circumference of the buttocks, ensuring it was snug but without compressing the skin. The obtained values were interpreted according to Polish age- and sex-specific HC percentile charts. HC below the 90th percentile was considered normal, whereas values at or above the 90th percentile indicated increased hip adiposity [17].

Waist-to-height ratio (WHtR) was calculated by dividing waist circumference (cm) by height (cm). A WHtR value ≥ 0.5 was used as an established threshold indicating increased cardiometabolic risk in pediatric populations.

All anthropometric measurements were performed by a single trained assessor certified at ISAK Level 1, following standardized ISAK protocols. Intra-rater reliability was quantified using the technical error of measurement (TEM), which was 0.26% for stature, 0.41% for waist circumference, and 0.38% for hip circumference, indicating high measurement precision. As all measurements were conducted by the same assessor, inter-rater reliability was not applicable [19].

### 2.3. Body Composition Analysis

Body composition was assessed using bioelectrical impedance analysis (BIA) with a Tanita BC-420MA analyzer (Tanita Corporation, Tokyo, Japan) according to standard operating procedures. This non-invasive technique determines body composition by assessing the opposition of body tissues to a low-intensity alternating electric current. Measurements were taken in the standing position, with participants barefoot and wearing light indoor clothing, without additional adjustment for clothing weight. In children with severe or profound intellectual disabilities, caregivers or teachers assisted when necessary, and sufficient time and repeated instructions were provided to minimize movement during assessment.

All measurements were conducted in the morning hours under standardized conditions. Participants and their parents or legal guardians were instructed to refrain from vigorous physical activity on the day of assessment and to attend the examination after an overnight fast (≥8 h). Measurements were performed in a temperature-controlled indoor environment, and assessments were scheduled within a consistent morning time window. Due to the specific characteristics of children with ID, information on voiding prior to measurement was obtained verbally from the child and/or caregiver when feasible, but could not be systematically verified. This approach was adopted to minimize the influence of recent food or fluid intake on body composition estimates [20]. The parameters obtained included body fat percentage (BF%), muscle mass percentage (MM%), fat-free mass percentage (FFM%) and total body water percentage (TBW%). According to age- and sex-specific percentile reference values for Polish children, participants were classified as non-obese (<95th percentile) or obese (≥95th percentile) [13].

### 2.4. Blood Pressure Measurement

BP measurements were obtained in the morning hours in a quiet room, following at least five minutes of seated rest. Each participant was seated comfortably with the back supported, feet flat on the floor, and the right arm positioned at heart level. We used a validated pediatric oscillometric device to take three consecutive readings, spaced 2 min apart. BP was measured using a pediatric-validated oscillometric device (Welch Allyn Spot Vital Signs 4400, Welch Allyn, Inc., Milwaukee, WI, USA), which has been validated for use in children and adolescents. The device was calibrated according to the manufacturer’s recommendations at regular intervals during the study period (before each school measurement cycle). Appropriate cuff sizes were selected individually for each participant based on mid–upper arm circumference, ensuring correct cuff width and bladder length in accordance with pediatric guidelines. All BP measurements were performed by the same trained investigator following a standardized protocol; therefore, inter-observer variability was not applicable. The average of the second and third readings was calculated for SBP and DBP, and this mean BP was used for analysis. BP percentiles were determined according to age-, sex-, and height-specific reference values recommended by the European Society of Hypertension, using the HyperChildNET Pediatric Blood Pressure Calculator—https://hyperchildnet.eu/blood-pressure-calculator/ (accessed on 22 January 2026).

BP categories were determined according to the 2016 European Society of Hypertension guidelines for children and adolescents, taking into account age-specific criteria. For participants younger than 16 y, classification was based on sex-, age-, and height-adjusted percentiles, whereas for those aged 16 y and older, fixed cut-off values recommended for adolescents and adults were used.

Accordingly, four BP categories were defined for the purposes of this study: Normal BP: systolic and/or diastolic BP < 90th percentile for children under 16 y, or <130/85 mmHg for participants aged 16 y and older. High-normal BP: SBP and/or DBP ≥ 90th to <95th percentile for children under 16 y, or 130–139/85–89 mmHg for those ≥16 y. Hypertension: SBP and/or DBP ≥ 95th percentile for children under 16 y, or ≥140/90 mmHg for those ≥6 y. Isolated systolic hypertension (ISH): SBP ≥ 95th percentile (or ≥140 mmHg for those ≥16 y) combined with diastolic pressure < 90th percentile (or <90 mmHg for those ≥16 y) [2]. BP classification followed the 2016 European Society of Hypertension guidelines, which apply percentile-based thresholds for children under 16 years of age and fixed cut-off values for adolescents aged 16 years and older, reflecting current clinical practice. Given that BP was assessed during a single visit, outcome categories should be interpreted as HTN-range/ISH-range BP at one occasion rather than clinically confirmed HTN.

Information obtained from the parental questionnaire indicated that four participants were receiving antihypertensive medication. As per the guideline recommendations, we classified these children as hypertensive regardless of their on-site BP readings.

### 2.5. Socio-Demographic Characteristics

Socio-demographic information was obtained using a structured questionnaire completed by parents or legal guardians. Data included the child’s sex, age, and degree of ID. Age was calculated from the date of birth provided by parents and verified with school records. For analysis, participants were categorized into two age groups: 7–12 y (younger children) and 13–18 y (adolescents). Sex was recorded as female or male, and the degree of ID was classified as mild, moderate, or severe/profound based on medical and educational documentation.

### 2.6. Ethics

Written informed consent was obtained from the parents or legal guardians of all participants, and verbal assent was obtained from the participating children, before enrollment in the study. The study was approved by the Bioethics Committee of the University of Rzeszów Decision No. 10/04/2019, issued on 11 April 2019) and conducted in accordance with the ethical principles outlined in the Declaration of Helsinki.

### 2.7. Statistical Analysis

Descriptive statistics are presented as mean ± standard deviation (M ± SD), median with interquartile range (Me ± IQR), minimum–maximum values, or number (percentage), as appropriate. Between-group differences in categorical variables were assessed using the chi-square test or the Freeman–Halton extension of Fisher’s exact test for larger contingency tables. Continuous variables were compared between two groups with the Mann–Whitney U test and across BP categories using the Kruskal–Wallis H test. Associations between BP and anthropometric/body composition parameters were examined with Spearman’s rank correlation coefficient.

We used multivariable logistic regression (stepwise) to examine variables associated with hypertension (HTN + ISH). Stepwise selection was used solely to reduce model dimensionality and limit overparameterization in exploratory subgroup analyses. The outcome was defined as HTN (including ISH) vs. normal BP (children with high-normal BP were excluded from this analysis). Regression models were exploratory and association-based; therefore, calibration metrics were not assessed, as no clinical prediction model was developed. Continuous predictor variables were dichotomized at ROC-derived exploratory cut-off values. We built separate regression models for the total sample and for subgroups stratified by sex and age. We evaluated each model’s discrimination exploratorily using the area under the ROC curve (AUC). Results are reported as odds ratios (OR) with 95% confidence intervals. Percentile-based reference values were used solely for descriptive classification of nutritional status and body composition prevalence. In contrast, ROC analyses and corresponding cut-off points were applied exclusively for exploratory discrimination purposes and were not used to define prevalence or clinical categories. For regression analyses, participants with high-normal BP were excluded to allow for comparison between clearly normotensive individuals and those with HTN-range/ISH-range BP, thereby improving the interpretability of associations in this screening-oriented, single-visit context. We did not apply dimension-reduction techniques, as the study aimed to compare clinically interpretable anthropometric and body composition indicators rather than to derive composite constructs; this exploratory approach may increase model complexity but supports clinical interpretability. A two-sided *p* < 0.05 was considered statistically significant. During the preparation of this manuscript, the authors used Napkin AI for the generation of figures. The authors reviewed and edited all generated materials and take full responsibility for the content, accuracy, and integrity of this publication.

## 3. Results

Table 1 presents the characteristics of the study sample by sex and age group. The study included 461 children and adolescents with ID aged 7–18 y, of whom 162 (35.1%) were girls and 299 (64.9%) were boys. The majority of participants (66.6%) were adolescents aged 13–18 y. Most children resided in rural areas (67.0%), and moderate ID was the most prevalent degree (49.5%), followed by mild (36.7%) and severe/profound (13.9%) disability.

Abdominal obesity was observed in 39.5% of participants, with a significantly higher prevalence among girls than boys (49.4% vs. 34.1%). Increased HC was found in 28.2% of the total sample, also more common in girls (34.6%) than in boys (24.7%).

Based on BP classification, 63.8% of participants had normal BP, 11.9% had high-normal BP, 13.9% were classified as having HTN, and 10.4% as having ISH. The combined prevalence of HTN and ISH was higher among girls than boys (31.4% vs. 20.4%) and in the older age group (13–18 y) compared to younger participants (25.1% vs. 22.7%). These proportions reflect HTN-range and ISH-range BP recorded at a single visit.

Regarding nutritional status, overweight and obesity based on BMI were identified in 28.2% and 17.4% of participants, respectively, with obesity being more common among girls (24.1%) than boys (13.7%). Similarly, excessive BF% (overfat + obesity BF% categories) was found in 40.6% of participants, again more prevalent among girls (55.0%) than boys (32.8%; *p* = 0.0001).

Detailed descriptive statistics for anthropometric, BP, and body composition parameters by sex and age group are provided in Supplementary Tables S1 and S2, respectively

Table 2 presents the distribution of BP categories by sex and age group. Compared with girls, boys had higher odds of having normal BP (OR = 1.72; *p* = 0.0071) and lower odds of having ISH (OR = 0.42; *p* = 0.0043). When age was considered, adolescents (13–18 y) had higher odds of ISH compared with younger children (7–12 y; OR = 2.73; *p* = 0.0121), whereas the odds of normal BP, high-normal BP, and HTN did not differ significantly between age groups.

Table 3 presents the distribution of BP categories across levels of ID. The distribution of BP categories differed significantly according to degree of ID (Freeman–Halton test: FFH(6) = 27.988; *p* = 0.0001). Using mild ID as the reference category, participants with moderate ID showed lower odds of high-normal BP (OR = 0.48; *p* = 0.0203) and higher odds of HTN (OR = 1.83; *p* = 0.0407). In contrast, individuals with severe or profound ID demonstrated markedly higher odds of ISH (OR = 4.87; *p* = 0.0003).

Table 4 presents the anthropometric and body composition parameters across BP categories. Significant differences were observed for all parameters. Higher BP categories were characterized by progressively greater values of WC, HC, BMI, and BF%, along with lower proportions of MM%, FFM%, and TBW%. Spearman correlation analyses showed moderate to strong positive associations between BP category and adiposity indicators (WC, BMI, BF%; r = 0.524–0.591; all *p* < 0.0001), and moderate to strong inverse associations with MM%, FFM%, and TBW% (r = –0.577 to –0.591; all *p* < 0.0001).

Detailed descriptive statistics for anthropometric, BP, and body composition parameters across BP categories, stratified by sex and age group, are presented in Supplementary Tables S3 and S4, respectively.

To derive exploratory cut-points differentiating participants with HTN-range/ISH-range BP (single visit) from those with normal BP, ROC analysis was performed. AUC values ranged from 0.575 to 0.844, indicating limited to good discriminatory accuracy for all anthropometric and body composition parameters. The highest AUC values were observed for HC (cut-off = 81.65 cm; AUC = 0.844), MM% (cut-off = 74.12%; AUC = 0.806), BF% (cut-off = 21.80%; AUC = 0.805), and FFM% (cut-off = 78.20%; AUC = 0.805). In contrast, WHtR showed limited discriminatory performance (cut-off = 0.5; AUC = 0.575), indicating weaker separation between participants with elevated BP and those with normal BP (Table 5).

Exploratory multivariable logistic regression models were used to examine factors associated with elevated BP (HTN-range/ISH-range) versus normal BP. All analyses were cross-sectional, and continuous predictors were dichotomized using ROC-derived cut-off points. In the total sample, higher WHtR ≥ 0.5 was associated with increased odds of elevated BP (OR = 4.25; *p* < 0.0001), whereas higher TBW% was inversely associated with elevated BP (OR = 0.17; *p* < 0.0001). Sex-stratified analyses showed that in girls, elevated BP was associated with higher HC and WHtR, while in boys, higher WHtR was associated with increased odds of elevated BP, and higher TBW% was inversely associated. In age-stratified analyses, higher TBW% was inversely associated with elevated BP in children aged 7–12 years. Among adolescents aged 13–18 years, higher WHtR was strongly associated with elevated BP, while higher TBW% showed an inverse association; BMI demonstrated a borderline association with elevated BP (Table 6).

## 4. Discussion

The present study of 461 children and adolescents with ID aged 7–18 y found a remarkably high burden of HTN and ISH in this underserved population: approximately 13.9% had HTN and 10.4% had ISH, giving a combined prevalence (HTN + ISH) of about 24.3%. Indicators of adiposity were also alarmingly prevalent (39.5% had abdominal obesity; 28.2% had an elevated HC), with girls more affected than boys in both measures (e.g., 49.4% vs. 34.1% for abdominal obesity). We observed that BP status was strongly associated with body composition: children in higher BP categories tended to have greater adiposity and lower muscle mass and lower TBW%. For example, BP category showed moderate-to-strong correlations with WC, BMI, and BF% (Spearman r ~0.53–0.59) and equally strong inverse correlations with MM%, FFM%, and TBW% (r ~−0.58; all *p* < 0.0001). Of all measures tested, HC was the best single discriminator of HTN + ISH (cut-off ~81.7 cm; AUC 0.844), outperforming BMI and all other body composition metrics. These cut-offs should be interpreted as exploratory and population-specific, rather than clinically validated thresholds. Accordingly, sensitivity and specificity estimates were not reported, as the present analyses were not designed to establish clinical decision thresholds and could otherwise overstate diagnostic or screening utility. Although HC showed the highest discriminatory performance among the assessed anthropometric measures, this finding should be interpreted cautiously. HC is strongly correlated with overall body size and adiposity and may therefore reflect global somatic growth rather than an independent risk marker. As the present analyses were not designed to assess incremental value beyond WC or BMI, the observed superiority of HC should be considered comparative rather than additive. Further studies are needed to determine whether HC provides independent value beyond established anthropometric indicators.

In exploratory multivariable logistic regression models, WHtR ≥ 0.5 was consistently associated with increased odds of elevated BP in the total sample, while higher TBW% showed a strong inverse association. In sex-stratified analyses, higher WHtR was associated with elevated BP in both girls and boys, whereas higher TBW% was inversely associated with elevated BP, particularly among boys. In age-stratified models, higher TBW% was inversely associated with elevated BP in children aged 7–12 years, while among adolescents (13–18 y) WHtR showed a strong positive association with elevated BP; BMI demonstrated only a borderline association in this age group. In addition, the distribution of BP categories varied significantly across levels of ID, with children with moderate ID showing higher odds of HTN and those with severe/profound ID showing markedly higher odds of ISH compared with other ID levels. These results suggest that in children with ID, not only adiposity, particularly central adiposity reflected by WHtR, but also unfavorable body-composition features such as lower TBW% (a BIA-derived proxy of lean tissue proportion) are associated with elevated BP.

The prevalence of HTN in children and adolescents with ID in our study exceeds that typically reported in general European pediatric populations, where a recent systematic review of 179,279 participants from 18 countries estimated the prevalence of HTN at 4% (95% CI 3–5%) [1]. For example, Lin et al. reported that 11.7% of Taiwanese adolescents with ID met the criteria for HTN [21], while Sun et al. observed a HTN prevalence of 31.4% in Chinese children with ID [4]. Moreover, population-based registry data from Denmark have demonstrated that individuals with ID have significantly elevated risks of cardiovascular disease across their life course—including hypertensive disease—with a hazard ratio of 1.30 (95% CI 1.22–1.39); risk increased with degree of ID [22]. This substantially higher burden underscores the vulnerability of the ID population to cardiovascular risk. Our findings corroborate this heightened risk and extend it by identifying specific anthropometric and body-composition correlates of elevated BP in this group.

The higher prevalence of HTN and ISH observed among girls in our study may be explained by several interrelated factors. Girls with intellectual disabilities may experience sex-specific pubertal and adiposity-related changes, which are associated with hormonal fluctuations and increased fat mass—both known contributors to elevated blood pressure [4]. Some studies, including mixed adolescent and adult samples, have reported higher obesity rates in females with mild to moderate intellectual disability, potentially further amplifying cardiovascular risk [23]. In addition, sex-related differences in stress reactivity or contextual factors during blood pressure measurement may partly influence systolic readings in adolescent girls [24].

Anthropometric indices such as WC and HC have been widely studied in typically developing children as predictors of elevated BP. For example, in a Taiwanese study of schoolchildren, elevated WC was independently associated with elevated BP, and HC was shown to be “as good as WC” in predicting elevated BP [10]. In a Lithuanian sample of adolescents aged 12–15 y, Kuciene and Dulskiene found that BMI and WC were stronger predictors of high BP than waist-to-height ratio [25]. Our finding that HC provided the highest AUC (0.844) for the discriminating of HTN + ISH in a pediatric population with ID supports the additional value of hip measurement—perhaps reflecting the distribution of adipose tissue or lean mass in the gluteofemoral region that may be particularly relevant in this population. Furthermore, the strong inverse associations of MM %, FFM % and TBW % with BP categories observed in descriptive and correlation analyses highlight that in children with ID, lower lean tissue proportion may compound the risk associated with excess adiposity.

These observations align with emerging work on body composition among children and adolescents with ID. In the study by Ungurean et al., the mean BMI values exceeded the thresholds recommended by the World Health Organization in both children with intellectual disabilities (ID) and their typically developing peers. The body-composition analysis revealed differences in anthropometric and morphological profiles between the groups, although the authors did not explicitly report that the prevalence of overweight or obesity was significantly higher in the ID group [26]. Similarly, Bertapelli et al. developed and cross-validated an easy-to-apply anthropometric equation for estimating BF% from BMI, age and sex in children with ID, demonstrating that simple field measures may provide valid estimates of adiposity for population-level screening in this specific population [14]. Furthermore, Ramos-Jiménez et al. developed combination metabolomic indices (anthropometric + biochemical) and found that they were associated with metabolic syndrome in adolescents and young adults with ID, suggesting that adiposity-related pathways may play a key role in cardiometabolic risk in this population [27]. Together with our findings, these data suggest that anthropometric and body-composition measures may offer scalable and informative tools for identifying cardiometabolic risk in pediatric populations with ID.

Mechanistically, excess visceral and abdominal fat may lead to heightened activation of the sympathetic nervous system, insulin resistance, endothelial dysfunction and low-grade inflammation, all of which can raise BP [8]. In children with ID, many of these pathways may be accentuated by reduced physical activity, higher sedentary time and impaired muscle development, although direct data in this population are scarce. The observed association in our study between greater muscle mass and lower odds of HTN + ISH is supported by findings in typically developing children showing that muscle mass was the strongest somatic-growth indicator associated with BP values in children and adolescents [9]. Furthermore, BIA-derived TBW% estimates have been shown to vary significantly with body composition in pediatric populations [28], although its direct link with BP in children remains less well-established. In this study, TBW% derived from bioelectrical impedance analysis should be interpreted as a body composition-related indicator reflecting lean tissue proportion rather than as a direct measure of hydration status. This interpretation is supported by the consistent inverse associations observed for TBW% as well as other lean mass-related parameters (FFM% and MM%) across BP categories. It should be noted that the use of a single-frequency BIA device limits detailed assessment of body water compartments; therefore, TBW% should be viewed as part of the overall body composition profile rather than a precise hydration marker.

From a clinical and public-health perspective, simple anthropometric measures (HC and WC), complemented by BIA where feasible, may support exploratory identification of children with elevated BP at a single visit and help prioritize further clinical assessment. As children with ID are often under-represented in routine preventive screening programs and have limited access to targeted health interventions, such screening could trigger earlier lifestyle counselling, physical-activity promotion and nutritional support. The fact that BMI and other commonly used indicators do not fully capture variability in BP is supported by the higher discriminatory performance of HC in exploratory ROC analyses and by the consistent associations observed for WHtR and TBW% in multivariable models, underscoring the need for more tailored, exploratory approaches in special-education settings. Additionally, our findings underline the urgency of implementing preventive strategies in children with ID: schools and care facilities should prioritize physical-activity programs tailored for cognitive and motor limitations, include muscle-strengthening exercises, and monitor body composition as well as BP.

This study has several important strengths. First, it is one of the largest investigations to date examining anthropometric and body-composition correlates of elevated BP in children and adolescents with ID. The inclusion of 461 participants across a wide age range provides robust statistical power and enhances the reliability of the observed associations. Second, all measurements were collected using standardized procedures by trained researchers in a controlled school setting, ensuring a high level of methodological consistency. Third, the study combined traditional anthropometric indicators (WC, HC, BMI) with detailed body-composition parameters obtained through BIA. This multimodal approach allowed for a more nuanced assessment of adiposity and muscularity than is typically available in research involving children with ID. Fourth, the use of ROC-derived thresholds and multivariable regression modeling provided data-driven insights into the discriminatory ability of different anthropometric and body-composition parameters for differentiating elevated BP categories. Finally, the inclusion of participants with varying degrees of ID enabled the examination of differences across these levels, offering new perspectives on how cognitive impairment may be associated with cardiovascular risk profiles.

This study has several important limitations. First, its cross-sectional design precludes conclusions regarding temporality or causality. Blood pressure was assessed during a single study visit; although three measurements were obtained using a standardized protocol and the average of the second and third readings was used to reduce measurement variability and first-measurement effects, sustained hypertension could not be confirmed. The lack of repeated measurements across multiple visits or ambulatory blood pressure monitoring may have led to an overestimation of elevated BP due to white-coat effects or day-to-day variability. Accordingly, the findings should be interpreted as indicators of elevated BP at a single visit rather than clinically confirmed hypertension.

Second, the study sample was drawn from special education schools in one region of Poland, which may limit generalizability. Although all eligible schools were invited, only seven participated, as recruitment coincided with the 2019–2020 COVID-19 pandemic and periods of strict lockdown that substantially restricted school availability. Participating schools were publicly funded institutions serving local catchment areas and included both urban and rural settings; however, selection bias at the school level cannot be excluded, and differences between participating and non-participating schools could not be formally assessed. Due to the limited number of schools, formal multilevel modeling was not performed, as estimates from mixed-effects models may be unstable with a small number of clusters.

Third, several potential confounding factors were not available, including pubertal stage, dietary patterns, physical activity, sleep-related disorders, medication use, and broader socioeconomic context. These factors may influence both body composition and blood pressure, particularly during adolescence, and their absence may have contributed to residual confounding. In addition, a small number of participants were unable to complete the BIA assessment due to inability to stand or high anxiety and may therefore differ systematically from those examined; as baseline data on excluded participants were not available, selection bias cannot be excluded.

Fourth, a small number of participants (n = 5) with medical conditions directly affecting blood pressure or hydration were excluded, which may have marginally underestimated hypertension prevalence. Due to the very small number of treated participants, separate sensitivity analyses excluding these individuals were not performed.

Finally, ROC-derived cut-off points were identified and applied within the same study sample and subsequently used in regression analyses, which may have introduced optimism bias and overestimated discriminatory performance and effect sizes. Therefore, these findings should be interpreted as exploratory and hypothesis-generating rather than confirmatory. Anthropometric indices and BIA-derived body composition parameters are inherently interrelated, and collinearity may have influenced regression coefficients and subgroup results. Although regression models were intentionally restricted in scope, residual collinearity cannot be excluded. In addition, multiple statistical comparisons were performed across correlated variables and subgroups without formal correction for multiple testing; accordingly, the results should be interpreted cautiously.

Future research should adopt longitudinal designs to assess how changes in body composition and anthropometric indices over time (including muscle mass gain, fat-mass reduction and changes in TBW%-related estimates) influence BP trajectories and sustained elevated BP in children with ID. Interventional studies are needed that specifically examine the impact of resistance-training programs, optimization of body composition and combined nutrition/physical-activity interventions on body composition, BP and vascular outcomes in this population. Additionally, expanding screening and research efforts to a wider geographic range and diverse populations of children with ID will improve external validity and help identify potential cultural or regional modifiers of risk. Further studies including external validation and cost-effectiveness or decision-analytic evaluations are needed before any widespread implementation of these anthropometric screening approaches can be recommended.

## 5. Conclusions

In this large cross-sectional study of Polish children and adolescents with ID, elevated BP—including HTN-range and ISH–range values assessed at a single visit—was highly prevalent. Anthropometric indicators of adiposity, particularly HC and WHtR, as well as body composition parameters reflecting higher fat mass and lower lean tissue proportion, were consistently associated with elevated BP categories. HC demonstrated the strongest discriminatory performance among the assessed measures; however, these findings should be interpreted as exploratory and population-specific rather than as validated screening thresholds. Overall, simple anthropometric and body composition assessments may support the identification of children with elevated BP in school-based settings, but do not constitute diagnostic or predictive tools.

Importantly, longitudinal studies with repeated BP measurements and external validation are required to confirm these associations, evaluate temporal relationships, and determine whether such measures can be meaningfully incorporated into preventive strategies for children and adolescents with ID.

## Figures and Tables

**Table 1:** Characteristics of the study sample by sex and age group.

Variable		Total		Girls		Boys		*p*	7–12 y		13–18 y		*p*
		n	%	n	%	n	%		n	%	n	%	
Age	7–12 y	154	33.4	48	29.6	106	35.5	0.2058	-				
	13–18 y	307	66.6	114	70.4	193	64.5						
Sex	Girls	162	35.1	-					48	31.2	114	37.1	0.2058
	Boys	299	64.9						106	68.8	193	62.9	
Place of residence	Rural area	309	67.0	117	72.2	192	64.2	0.0808	90	58.4	219	71.3	**0.0055**
	Urban area	152	33.0	45	27.8	107	35.8		64	41.6	88	28.7	
Degree of ID	Mild	169	36.7	60	37.0	109	36.5	0.9868	70	45.5	99	32.2	**<0.0001**
	Moderate	228	49.5	80	49.4	148	49.5		78	50.6	150	48.9	
	Severe (n = 62)/Profound (n = 1)	63	13.9	22	13.6	42	14.0		6	3.9	58	18.9	
WC	Normal WC	279	60.5	82	50.6	197	65.9	**0.0014**	97	63.0	182	59.3	0.4429
	Abdominal obesity	182	39.5	80	49.4	102	34.1		57	37.0	125	40.7	
HC	Normal HC	331	71.8	106	65.4	225	75.3	**0.0253**	112	72.7	219	71.3	0.7541
	High HC	130	28.2	56	34.6	74	24.7		42	27.3	88	28.7	
BP classification	Normal	294	63.8	90	55.6	204	68.2	**0.0121**	101	65.6	193	62.9	**0.0385**
	High-normal	55	11.9	21	13.0	34	11.4		18	11.7	37	12.1	
	HTN	64	13.9	25	15.4	39	13.0		27	17.5	37	12.1	
	ISH	48	10.4	26	16.0	22	7.4		8	5.2	40	13.0	
BMI category	Underweight	34	7.4	12	7.4	22	7.4	**0.0167**	9	5.8	25	8.1	**0.0127**
	Normal weight	217	47.1	63	38.9	154	51.5		89	57.8	128	41.7	
	Overweight	130	28.2	48	29.6	82	27.4		36	23.4	94	30.6	
	Obesity	80	17.4	39	24.1	41	13.7		20	13.0	60	19.5	
BF% category	Underfat	89	19.3	14	8.6	75	25.1	**0.0001**	25	16.2	64	20.8	0.0722
	Normal	185	40.1	59	36.4	126	42.1		72	46.8	113	36.8	
	Overfat	69	15.0	28	17.3	41	13.7		16	10.4	53	17.3	
	Obesity	118	25.6	61	37.7	57	19.1		41	26.6	77	25.1	

Data are presented as number (percentage). *p*-values refer to group comparisons based on the chi-square test (χ^2^). Bolded *p*-values indicate statistically significant differences. Abbreviations: WC—waist circumference; HC—hip circumference; BMI—body mass index; BF%—body fat percentage; BP—blood pressure; HTN—hypertension; ISH—isolated systolic hypertension; ID—intellectual disability.

**Table 2:** Distribution of blood pressure categories by sex and age group.

BP Classification	Total		Girls		Boys		OR (95% CI)	7–12 y		13–18 y		OR (95% CI)
	n	%	n	%	n	%		n	%	n	%	
Normal	294	63.8	90	55.6	204	68.2	**1.72 (1.16–2.55); *p* = 0.0071**	101	65.6	193	62.9	0.89 (0.59–1.33); *p* = 0.5670
High-normal	55	11.9	21	13.0	34	11.4	0.86 (0.48–1.54); *p* = 0.6150	18	11.7	37	12.1	1.04 (0.57–1.89); *p* = 0.9095
HTN	64	13.9	25	15.4	39	13.0	0.82 (0.48–1.41); *p* = 0.4793	27	17.5	37	12.1	0.64 (0.38–1.11); *p* = 0.1103
ISH	48	10.4	26	16.0	22	7.4	**0.42 (0.23–0.76); *p* = 0.0043**	8	5.2	40	13.0	**2.73 (1.25–6.0); *p* = 0.0121**
*p*	**χ^2^(3) = 10.924; *p* = 0.0121**						*p*	**χ^2^(3) = 8.394; *p* = 0.0385**				*p*

Data are presented as number (percentage). *p*-values refer to group comparisons based on the chi-square test (χ^2^). Odds ratios (ORs) are shown for boys compared with girls and for adolescents (13–18 y) compared with younger children (7–12 y). Statistically significant results are indicated in bold. Abbreviations: BP—blood pressure; HTN—hypertension; ISH—isolated systolic hypertension.

**Table 3:** Distribution of blood pressure categories across levels of intellectual disability.

BP Classification	Total		Mild (REF)		Moderate		OR (95% CI)	Severe/ Profound		OR (95% CI)
	n	%	n	%	n	%		n	%	
Normal	294	63.8	112	66.3	142	62.3	0.84 (0.55–1.27); *p* = 0.4129	40	62.5	0.85 (0.47–1.54); *p* = 0.5896
High-normal	55	11.9	28	16.6	20	8.8	**0.48 (0.26–0.89); *p* = 0.0203**	7	10.9	0.62 (0.26–1.50); *p* = 0.2864
HTN	64	13.9	19	11.2	43	18.9	**1.83 (1.03–3.28); *p* = 0.0407**	2	3.1	0.25 (0.06–1.13); *p* = 0.0714
ISH	48	10.4	10	5.9	23	10.1	1.78 (0.83–3.86); *p* = 0.1411	15	23.4	**4.87 (2.06–11.52); *p* = 0.0003**

Data are presented as number (percentage). Group differences were assessed using the Freeman–Halton extension of Fisher’s exact test (FFH). Odds ratios (OR) with 95% confidence intervals (CI) were estimated using mild ID as the reference category for each BP category. ORs represent cross-sectional associations and should not be interpreted as causal effects. Statistically significant results are indicated in bold. Abbreviations: BP—blood pressure; HTN—hypertension; ISH—isolated systolic hypertension.

**Table 4:** Anthropometric and body composition parameters across blood pressure categories.

Variable	BP Classification	Total			*p*	
		M ± SD	Me ± IQR	Min-Max		
WC (cm)	Normal	71.33 ± 13.83	70.00 ± 15.30	48.00–169.40	H = 128.244; ***p* < 0.0001**	R = 0.524; ***p* < 0.0001**
	High-normal	84.48 ± 14.23	84.00 ± 18.10	48.60–109.40		
	HTN	89.93 ± 17.07	87.00 ± 25.70	51.40–136.00		
	ISH	90.54 ± 15.26	91.75 ± 20.50	62.00–127.00		
HC (cm)	Normal	84.54 ± 12.31	85.30 ± 18.10	53.50–112.0	H = 100.167; ***p* < 0.0001**	R = 0.466; ***p* < 0.0001**
	High-normal	95.00 ± 15.23	95.00 ± 18.10	56.80–120.00		
	HTN	98.25 ± 14.80	98.50 ± 20.80	58.20–135.50		
	ISH	103.11 ± 12.47	104.70 ± 17.35	69.00–126.00		
BMI (kg/m^2^)	Normal	20.50 ± 4.15	20.15 ± 5.90	13.0–32.80	H = 153.242; ***p* < 0.0001**	R = 0.573; ***p* < 0.0001**
	High-normal	26.25 ± 5.54	25.80 ± 7.30	14.0–40.60		
	HTN	27.98 ± 5.70	27.35 ± 7.50	14.9–47.70		
	ISH	28.94 ± 6.20	28.35 ± 9.20	17.60–42.80		
BF%	Normal	17.91 ± 8.22	17.00 ± 11.00	3.00–43.80	H = 166.647; ***p* < 0.0001**	R = 0.591; ***p* < 0.0001**
	High-normal	28.88 ± 8.93	28.20 ± 13.10	5.20–46.10		
	HTN	32.35 ± 7.95	32.40 ± 8.75	14.50–52.10		
	ISH	31.66 ± 9.84	31.15 ± 15.10	9.70–51.20		
MM%	Normal	77.82 ± 7.78	78.71 ± 10.38	53.28–92.10	H = 166.109; ***p* < 0.0001**	R = −0.590; ***p* < 0.0001**
	High-normal	67.44 ± 8.41	68.27 ± 12.18	50.93–90.07		
	HTN	64.17 ± 7.55	64.13 ± 8.32	45.41–81.23		
	ISH	64.87 ± 9.34	65.35 ± 14.14	46.40–85.69		
FFM%	Normal	82.09 ± 8.22	83.00 ± 11.00	56.20–97.00	H = 166.647; ***p* < 0.0001**	R = −0.591; ***p* < 0.0001**
	High-normal	71.12 ± 8.93	71.80 ± 13.10	53.90–94.80		
	HTN	67.65 ± 7.95	67.60 ± 8.75	47.90–85.50		
	ISH	68.34 ± 9.84	68.85 ± 15.10	48.80–90.30		
TBW%	Normal	60.32 ± 6.46	60.86 ± 8.36	41.08–79.47	H = 159.686; ***p* < 0.0001**	R = −0.577; ***p* < 0.0001**
	High-normal	52.40 ± 6.57	52.59 ± 7.78	39.44–69.49		
	HTN	49.73 ± 5.73	49.51 ± 6.32	35.02–62.65		
	ISH	50.68 ± 6.89	50.37 ± 9.24	35.77–66.19		

Data are presented as mean ± standard deviation (M ± SD), median ± interquartile range (Me ± IQR), and minimum–maximum values. Differences between BP categories were assessed using the Kruskal–Wallis H test (H), and associations were evaluated using Spearman’s rank correlation coefficient (R). Statistically significant results are indicated in bold. Abbreviations: WC—waist circumference; HC—hip circumference; BMI—body mass index; BF%—body fat percentage; MM%—muscle mass percentage; FFM%—fat-free mass percentage; TBW%—total body water percentage; BP—blood pressure; HTN—hypertension; ISH—isolated systolic hypertension.

**Table 5:** Exploratory ROC-derived cut-off points and discriminatory performance of anthropometric and body composition parameters for elevated blood pressure (HTN + ISH).

Variable	Cut Point	n	%	AUC
WC (cm)	<64.45	98	21.3%	AUC = 0.754 (0.707–0.802); ***p* < 0.0001**
	≥64.45	363	78.7%	
HC (cm)	<81.65	123	26.7%	AUC = 0.844 (0.804–0.884); ***p* < 0.0001**
	≥81.65	338	73.3%	
BMI (kg/m^2^)	<19.75	152	33.0%	AUC = 0.729 (0.68–0.779); ***p* < 0.0001**
	≥19.75	309	67.0%	
BF%	<21.80	232	50.3%	AUC = 0.805 (0.763–0.847); ***p* < 0.0001**
	≥21.80	229	49.7%	
MM%	<74.12	229	49.7%	AUC = 0.806 (0.764–0.847); ***p* < 0.0001**
	≥74.12	232	50.3%	
FFM%	<78.20	229	49.7%	AUC = 0.805 (0.763–0.847); ***p* < 0.0001**
	≥78.20	232	50.3%	
TBW%	<55.38	191	41.4%	AUC = 0.801 (0.759–0.843); ***p* < 0.0001**
	≥55.38	270	58.6%	
WHtR	<0.5	261	56.6%	AUC = 0.575 (0.520–0.631); ***p* = 0.0076**
	≥0.5	200	43.4%	

Data are presented as absolute numbers (n) and percentages (%) of participants below and above each ROC-derived cut-off point. The area under the ROC curve (AUC) with 95% confidence intervals (CI) reflects the exploratory ability of each parameter to discriminate participants with elevated BP (HTN-range/ISH-range) at a single visit from those with normal BP; individuals with high-normal BP were excluded from this comparison. Statistically significant results are indicated in bold. Abbreviations: WC—waist circumference; HC—hip circumference; BMI—body mass index; BF%—body fat percentage; MM%—muscle mass percentage; FFM%—fat-free mass percentage; TBW%—total body water percentage; WHtR—waist-to-height ratio; HTN—hypertension; ISH—isolated systolic hypertension.

**Table 6:** Exploratory multivariable logistic regression models of factors associated with elevated BP (HTN-range/ISH-range).

Group	Variable	B/SE	Wald	OR (95% CI)
Total (n = 461)	TBW%	B = −1.79 ± 0.31	34.46	**OR = 0.17 (0.09–0.30); *p* < 0.0001**
	WHtR	B = 1.45 ± 0.31	21.22	**OR = 4.25 (2.30–7.87); *p* < 0.0001**
Girls (n = 162)	HC	B = 1.85 ± 0.80	5.32	**OR = 6.38 (1.32–30.80); *p* = 0.0210**
	WHtR	B = 2.12 ± 0.46	21.16	**OR = 8.33 (3.38–20.55); *p* < 0.0001**
Boys (n = 299)	TBW%	B = −2.14 ± 0.39	29.66	**OR = 0.12 (0.05–0.25); *p* < 0.0001**
	WHtR	B = 1.27 ± 0.43	8.82	**OR = 3.55 (1.54–8.21); *p* = 0.0030**
7–12 y (n = 154)	TBW%	B = −3.27 ± 0.57	32.46	**OR = 0.04 (0.01–0.12); *p* < 0.0001**
13–18 y (n = 307)	BMI	B = 1.96 ± 1.06	3.45	OR = 7.11 (0.90–56.45); *p* = 0.0634
	TBW%	B = −1.27 ± 0.34	13.61	**OR = 0.28 (0.14–0.55); *p* = 0.0002**
	WHtR	B = 1.61 ± 0.37	18.85	**OR = 5.01 (2.42–10.36); *p* < 0.0001**

Data are presented as odds ratios (OR) with 95% confidence intervals (CI) derived from exploratory multivariable logistic regression models. Continuous predictors were dichotomized using ROC-derived cut-off points for exploratory purposes only. Regression models were constructed separately for the total sample and for subgroups stratified by sex and age. Statistically significant results are indicated in bold. Abbreviations: WHtR—waist-to-height ratio; TBW%—total body water percentage (BIA-derived proxy related to lean tissue proportion); BMI—body mass index; BP—blood pressure; HTN—hypertension; ISH—isolated systolic hypertension.

## Data Availability

The data presented in this study are available on reasonable request from the corresponding author.

## Supplementary Materials

The following supporting information can be downloaded at: https://www.mdpi.com/article/10.3390/jcm15031058/s1. Table S1: Detailed descriptive statistics for anthropometric, BP, and body composition parameters by sex in the total study sample; Table S2: Detailed descriptive statistics for anthropometric, BP, and body composition parameters by age group; Table S3: Anthropometric, blood pressure, and body composition parameters across blood pressure categories, stratified by sex (girls and boys); Table S4: Anthropometric, blood pressure, and body composition parameters across blood pressure categories, stratified by age group (7–12 y and 13–18 y).

## Abbreviations

The following abbreviations are used in this manuscript:

ID	Intellectual disability
BP	Blood pressure
HTN	Hypertension
ISH	Isolated systolic hypertension
WC	Waist circumference
HC	Hip circumference
BMI	Body mass index
BF%	Body fat percentage
MM%	Muscle mass percentage
FFM%	Fat-free mass percentage
TBW%	Total body water percentage
AUC	Area under the curve
ROC	Receiver operating characteristic
OR	Odds ratio
CI	Confidence interval
