# Supplementary material for: Anthropometric and Body Composition Correlates of Hypertension in Children and Adolescents with Intellectual Disabilities
Source: J Clin Med. 2026 Jan 28;15(3):1058. doi: 10.3390/jcm15031058 (PMC12897980; doi:10.3390/jcm15031058)
Supplement: Supplementary file 1 [file jcm-15-01058-s001.zip › jcm-4049145-supplementary.pdf]

Supplementary Table S1. Detailed descriptive statistics for anthropometric, BP, and body composition parameters by sex in the total study sample.

Total	M±SD	Me±IQR	Min-Max	Sex	M±SD	Me±IQR	Min-Max	p
Height (cm)	154.91±17.04	156.5±23.40	96-191.50	girls	150.16±14.05	152.40±19.20	104.50-184.00	**Z=-5.067; p<0.0001**
				boys	157.48±17.97	160.30±25.50	96.00-191.50	
Age (years)	14.15±3.50	15.00±7.00	7.00-18.00	girls	14.48±3.51	16.00±6.00	7.00-18.00	Z=-1.525; p=0.1272
				boys	13.96±3.49	14.00±7.00	7.00-18.00	
WC (cm)	77.48±16.69	75.00±21.50	48.00-169.40	girls	78.19±18.53	75.80±22.00	48.00-169.40	Z=-0.432; p=0.6660
				boys	77.09±15.63	74.60±21.00	49.00-127.00	
HC (cm)	89.62±14.80	89.00±18.10	53.50-135.50	girls	92.51±15.55	92.00±20.50	57.00-135.50	**Z=-2.830; p=0.0047**
				boys	88.06±14.16	88.30±18.00	53.50-125.00	
SBP (mmHg)	121.18±14.57	117.00±17.00	71.00-186.00	girls	124.00±14.92	122.00±21.00	77.00-162.00	**Z=-3.636; p=0.0003**
				boys	119.65±14.17	116.00±13.50	71.00-186.00	
DBP (mmHg)	69.63±10.28	69.00±12.00	46.00-116.00	girls	71.55±11.48	69.00±13.00	46.00-113.00	**Z=-2.399; p=0.0164**
				boys	68.59±9.42	68.00±12.00	47.00-116.00	
BMI	23.10±5.94	22.20±8.10	13.00-47.70	girls	24.50±6.57	23.70±8.40	13.00-47.70	**Z=-3.381; p=0.0007**
				boys	22.35±5.44	21.30±8.10	13.10-42.50	
BF%	22.65±10.56	21.70±15.70	3.00-52.10	girls	29.97±9.68	31.15±13.80	4.00-52.10	**Z=-10.824; p<0.0001**
				boys	18.69±8.75	17.50±12.10	3.00-47.80	
Body fat mass (kg)	13.97±10.08	11.90±13.40	0.90-59.70	girls	18.42±11.08	16.20±15.60	0.90-59.70	**Z=-6.987; p<0.0001**
				boys	11.56±8.60	9.00±11.00	0.90-47.80	
Muscle mass (kg)	40.86±13.31	40.30±19.00	12.40-78.30	girls	36.11±9.21	37.05±12.4	13.50-59.60	**Z=-5.700; p<0.0001**
				boys	43.43±14.45	44.10±22.30	12.40-78.30	

MM%	73.34±9.99	74.33±14.81	45.41-92.10	girls	66.41±9.14	65.30±12.88	45.41-90.78	**Z=-10.842; p<0.0001**
				boys	77.09±8.29	78.14±11.42	49.28-92.10	
FFM (kg)	43.07±13.97	42.50±20.00	13.30-82.30	girls	38.05±9.67	39.05±13.10	14.30-62.80	**Z=-5.729; p<0.0001**
				boys	45.78±15.15	46.50±23.40	13.30-82.30	
FFM%	77.35±10.56	78.30±15.70	47.90-97.00	girls	70.03±9.68	68.85±13.80	47.90-96.00	**Z=-10.824; p<0.0001**
				boys	81.31±8.75	82.50±12.10	52.20-97.00	
TBW (kg)	31.66±10.21	31.80±14.80	9.70-60.50	girls	27.80±7.00	28.30±9.70	10.50-46.20	**Z=-6.140; p<0.0001**
				boys	33.73±11.03	35.10±17.75	9.70-60.50	
TBW%	56.90±7.88	57.39±11.48	35.02-79.47	girls	51.56±6.92	50.50±9.85	35.02-70.05	**Z=-10.679; p<0.0001**
				boys	59.79±6.78	60.41±9.31	38.16-79.47	

Data are presented as mean ± standard deviation (M±SD), median ± interquartile range (Me±IQR), and minimum–maximum values. Between-group differences were analyzed using the Mann–Whitney U test. Statistically significant results are indicated in bold. Abbreviations: BMI – body mass index; BF% – body fat percentage; MM – muscle mass; MM% – muscle mass percentage; FFM – fat-free mass; FFM% – fat-free mass percentage; TBW – total body water; TBW% – total body water percentage; SBP – systolic blood pressure; DBP – diastolic blood pressure.

Supplementary Table S2. Detailed descriptive statistics for anthropometric, BP, and body composition parameters by age group.

**Total**	**Age**	**M±SD**	**Me±IQR**	**Min-Max**	**p**
Height (cm)	7-12 y	138.87±14.06	140.40±18.60	96.00-168.00	**Z=-14.156; p<0.0001**
	13-18 y	162.96±11.97	163.30±18.00	131.20-191.50	
Age (years)	7-12 y	9.88±1.70	10.00±3.00	7.00-12.00	**Z=-17.723; p<0.0001**
	13-18 y	16.28±1.80	17.00±3.00	13.00-18.00	
WC (cm)	7-12 y	68.23±13.11	66.15±18	48.00-112.70	**Z=-8.913; p<0.0001**
	13-18 y	82.12±16.38	79.00±22.00	50.90-169.40	
HC (cm)	7-12 y	77.87±13.030	75.95±17.50	53.50-125.00	**Z=-12.041; p<0.0001**

	13-18 y	95.52±11.83	94.00±16.00	66.80-135.50	
SBP (mmHg)	7-12 y	115.67±12.96	113.50±14.00	85.00-186.00	**Z=-6.621; p<0.0001**
	13-18 y	123.94±14.58	119.00±20.00	71.00-179.00	
DBP (mmHg)	7-12 y	65.15±9.82	65.00±9.00	47.00-116.00	**Z=-8.223; p<0.0001**
	13-18 y	71.87±9.77	71.50±11.00	46.00-113.00	
BMI	7-12 y	20.15±4.91	18.85±7.00	13.00-34.20	**Z=-8.042; p<0.0001**
	13-18 y	24.59±5.87	23.80±7.70	13.10-47.70	
BF%	7-12 y	22.36±9.55	19.45±14.30	4.00-47.80	Z=-0.610; p=0.5418
	13-18 y	22.80±11.04	22.70±16.40	3.00-52.10	
Body fat mass (kg)	7-12 y	9.92±7.41	7.50±9.70	0.90-31.50	**Z=-6.322; p<0.0001**
	13-18 y	16.00±10.63	13.80±14.40	0.90-59.70	
Muscle mass (kg)	7-12 y	28.59±8.75	28.10±11.90	12.40-57.90	**Z=-14.480; p<0.0001**
	13-18 y	47.01±10.70	46.40±14.30	25.60-78.30	
MM%	7-12 y	73.46±8.97	75.99±13.58	49.28-90.78	Z=-0.351; p=0.7256
	13-18 y	73.28±10.47	73.38±15.58	45.41-92.10	
FFM (kg)	7-12 y	30.20±9.17	29.70±12.60	13.30-60.90	**Z=-14.472; p<0.0001**
	13-18 y	49.52±11.23	48.90±15.00	27.00-82.30	
FFM%	7-12 y	77.64±9.55	80.55±14.30	52.20-96.00	Z=-0.610; p=0.5418
	13-18 y	77.20±11.04	77.30±16.40	47.90-97.00	
TBW (kg)	7-12 y	22.15±6.79	21.70±9.60	9.70-44.60	**Z=-14.486; p<0.0001**
	13-18 y	36.50±8.02	36.10±11.00	19.80-60.50	
TBW%	7-12 y	56.94±7.26	58.80±10.52	38.16-79.47	Z=-0.257; p=0.7970
	13-18 y	56.88±8.18	56.60±12.35	35.02-76.62	

Data are presented as mean ± standard deviation (M±SD), median ± interquartile range (Me±IQR), and minimum–maximum values. Between-group differences were analyzed using the Mann–Whitney U test. Statistically significant results are indicated in bold. Abbreviations: BMI – body mass index; BF% – body fat percentage; MM – muscle mass; MM% – muscle mass percentage; FFM – fat-free mass; FFM% – fat-free mass percentage; TBW – total body water; TBW% – total body water percentage; SBP – systolic blood pressure; DBP – diastolic blood pressure.

Supplementary Table S3. Anthropometric, blood pressure, and body composition parameters across blood pressure categories, stratified by sex (girls and boys).

Variable	BP classification	Girls			p		Boys			p	
		M±SD	Me±IQR	Min-Max			M±SD	Me±IQR	Min-Max		
WC (cm)	Normal	71.64±18.95	69.40±16.60	48.00-169.40	**H=44.635; p<0.0001**	**R=0.500; p<0.0001**	71.19±10.89	70.20±16.00	49.00-99.00	**H=85.143; p<0.0001**	**R=0.533; p<0.0001**
	High-normal	86.49±14.02	85.00±13.20	48.60-109.40			83.23±14.42	83.40±21.00	49.50-107.40		
	HTN	84.86±15.51	80.00±13.70	60.20-136.00			93.17±17.42	96.00±33.30	51.40-120.20		
	ISH	87.76±13.86	85.00±18.00	63.10-115.00			93.83±16.47	94.75±17.00	62.00-127.00		
HC (cm)	Normal	86.17±13.30	86.70±17.00	57.00-112.00	**H=34.374; p<0.0001**	**R=0.439; p<0.0001**	83.81±11.81	85.00±18.10	53.50-107.00	**H=64.442; p<0.0001**	**R=0.459; p<0.0001**
	High-normal	100.27±16.04	103.00±26.40	58.70-120.00			91.74±13.96	90.65±18.5	56.80-120.00		
	HTN	97.68±14.81	96.00±21.40	71.30-135.50			98.62±14.98	99.00±21.40	58.20-125.00		
	ISH	103.20±13.06	102.70±22.00	81.30-126.00			103.00±12.05	105.00±15.00	69.00-122.50		
BMI (kg/m²)	Normal	21.15±4.60	20.85±6.40	13.00-32.80	**H=55.607; p<0.0001**	**R=0.561; p<0.0001**	20.22±3.91	19.70±5.40	13.10-29.90	**H=94.137; p<0.0001**	**R=0.559; p<0.0001**
	High-normal	28.61±5.69	29.40±4.60	16.40-40.60			24.79±4.99	25.15±4.80	14-36.70		
	HTN	28.18±6.85	25.30±8.20	18.3-47.70			27.85±4.91	29.40±7.30	14.90-36.10		
	ISH	29.25±6.32	28.70±9.00	17.60-42.80			28.57±6.18	28.35±8.90	18.40-42.50		
BF%	Normal	24.54±7.89	24.45±12.40	4.00-43.80	**H=66.245; p<0.0001**	**R=0.610; p<0.0001**	14.98±6.48	15.10±8.55	3.00-43.10	**H=117.415; p<0.0001**	**R=0.611; p<0.0001**
	High-normal	36.07±6.30	38.40±6.40	19.90-44.90			24.43±7.30	25.30±5.70	5.20-46.10		
	HTN	36.91±6.90	36.20±7.80	19.50-52.10			29.42±7.23	29.80±8.60	14.50-47.80		
	ISH	37.18±8.01	36.85±13	19.40-51.20			25.13±7.61	25.65±10.60	9.70-40.50		
MM%	Normal	71.55±7.43	71.66±11.51	53.28-90.78	**H=66.270; p<0.0001**	**R=-0.61; p=0.0001**	80.58±6.17	80.49±8.27	53.83-92.10	**H=116.878; p<0.0001**	**R=-0.609; p<0.0001**
	High-normal	60.66±5.90	58.49±6.04	52.28-75.42			71.63±6.88	70.89±5.29	50.93-90.07		
	HTN	59.83±6.53	60.34±7.12	45.41-76.33			66.95±6.87	66.61±8.09	49.28-81.23		
	ISH	59.61±7.59	59.90±12.29	46.40-76.54			71.08±7.20	70.43±10.09	56.53-85.69		
FFM%	Normal	75.46±7.89	75.55±12.40	56.20-96.00	**H=66.245; p<0.0001**	**R=-0.610; p<0.0001**	85.02±6.48	84.90±8.55	56.90-97.00	**H=117.415; p<0.0001**	**R=-0.611; p<0.0001**
	High-normal	63.93±6.30	61.60±6.40	55.10-80.10			75.57±7.30	74.70±5.70	53.90-94.80		
	HTN	63.09±6.90	63.80±7.80	47.90-80.50			70.58±7.23	70.20±8.60	52.20-85.50		
	ISH	62.82±8.01	63.15±13.00	48.80-80.60			74.87±7.61	74.35±10.60	59.50-90.30		

TBW%	Normal	55.23±5.91	55.27±8.93	41.08-70.05	**H=57.886; p<0.0001**	**R=-0.571; p<0.0001**	62.56±5.33	62.25±7.14	41.57-79.47	**H=114.030; p<0.0001**	**R=-0.598; p<0.0001**
	High-normal	47.30±5.14	46.52±6.10	40.34-58.66			55.54±5.29	55.31±4.32	39.44-69.49		
	HTN	46.76±5.15	46.91±5.66	35.02-58.97			51.64±5.31	51.53±6.28	38.16-62.65		
	ISH	46.91±5.36	47.89±8.54	35.77-59.05			55.15±5.78	54.61±7.71	43.56-66.19		

Data are presented as mean ± standard deviation (M±SD), median ± interquartile range (Me±IQR), and minimum–maximum values. Differences between blood pressure (BP) categories were assessed using the Kruskal–Wallis H test (H), and associations were evaluated using Spearman's rank correlation coefficient (R). Statistically significant results are indicated in bold. Abbreviations: WC – waist circumference; HC – hip circumference; BMI – body mass index; BF% – body fat percentage; MM% – muscle mass percentage; FFM% – fat-free mass percentage; TBW% – total body water percentage; BP – blood pressure; HTN – hypertension; ISH – isolated systolic hypertension.

Supplementary Table S4. Anthropometric, blood pressure, and body composition parameters across blood pressure categories, stratified by age group (7–12 y and 13–18 y).

**Variable**	**BP classification**	**7-12 y**			**p**		**13-18 y**			**p**	
		**M±SD**	**Me±IQR**	**Min-Max**			**M±SD**	**Me±IQR**	**Min-Max**		
WC (cm)	Normal	63.42±9.71	61.40±14.80	48.00-90.30	**H=42.311; p<0.0001**	**R=0.506; p<0.0001**	75.46±13.88	73.00±15.00	50.90-169.40	**H=107.365; p<0.0001**	**R=0.581; p<0.0001**
	High-normal	72.84±14.65	72.75±16	48.60-100.00			90.14±10.08	90.00±12.20	69.40-109.40		
	HTN	81.57±13.33	78.80±14.4	51.40-112.70			96.02±17.06	97.30±28.00	60.20-136.00		
	ISH	73.45±11.08	71±17.45	62.00-92.50			93.96±13.66	94.50±19.00	69.40-127.00		
HC (cm)	Normal	73.11±9.54	73.00±13.00	53.50-101.00	**H=39.752; p<0.0001**	**R=0.503; p<0.0001**	90.52±8.91	90.00±12.80	66.80-112.00	**H=88.993; p<0.0001**	**R=0.538; p<0.0001**
	High-normal	81.74±13.79	85.75±16.9	56.80-107.00			101.45±11.29	103.00±15.90	83.00-120.00		
	HTN	90.61±14.67	92.40±18.00	58.20-125.00			103.83±12.33	105.90±19.6	83.00-135.50		
	ISH	86.23±8.93	87.50±9.35	69.00-98.50			106.48±10.15	107.25±15.35	87.70-126.00		
BMI (kg/m^2)	Normal	17.98±3.20	17.30±4.00	13.00-30.60	**H=55.279; p<0.0001**	**R=0.588; p<0.0001**	21.83±3.97	21.30±5.70	13.10-32.80	**H=113.983; p<0.0001**	**R=0.602; p<0.0001**
	High-normal	22.46±4.77	22.70±7.20	14.00-32.50			28.10±4.96	27.40±5.90	18.80-40.60		
	HTN	25.79±4.72	25.30±6.90	14.90-34.20			29.58±5.88	29.40±6.70	18.30-47.70		
	ISH	23.40±5.08	22.05±8.15	17.60-31.40			30.05±5.84	29.90±8.65	21.50-42.80		

BF%	Normal	17.99±6.87	16.50±6.40	4.00-43.10	**H=62.949; p<0.0001**	**R=0.631; p<0.0001**	17.86±8.86	17.80±12.70	3.00-43.80	**H=104.594; p<0.0001**	**R=0.571; p<0.0001**
	High-normal	26.36±8.89	25.80±11.70	9.90-46.10			30.10±8.81	28.30±14.40	5.20-44.90		
	HTN	33.54±6.97	34.00±7.10	17.30-47.80			31.48±8.58	31.40±8.20	14.50-52.10		
	ISH	30.79±8.27	27.90±11.50	19.40-44.30			31.84±10.21	31.40±15.50	9.70-51.20		
MM%	Normal	77.56±6.46	78.95±6.48	53.83-90.78	**H=63.551; p<0.0001**	**R=-0.635; p<0.0001**	77.95±8.40	78.05±11.92	53.28-92.10	**H=104.336; p<0.0001**	**R=-0.570; p<0.0001**
	High-normal	69.66±8.29	70.35±10.65	50.93-85.06			66.36±8.37	67.93±13.62	52.28-90.07		
	HTN	62.96±6.57	62.66±6.61	49.28-78.40			65.06±8.17	65.07±7.77	45.41-81.23		
	ISH	65.62±7.77	68.40±10.56	52.94-76.54			64.72±9.70	65.12±14.75	46.40-85.69		
FFM%	Normal	82.01±6.87	83.50±6.40	56.90-96.00	**H=62.949; p<0.0001**	**R=-0.631; p<0.0001**	82.14±8.86	82.20±12.70	56.20-97.00	**H=104.594; p<0.0001**	**R=-0.571; p<0.0001**
	High-normal	73.64±8.89	74.20±11.70	53.90-90.10			69.90±8.81	71.70±14.40	55.10-94.80		
	HTN	66.46±6.97	66.00±7.10	52.20-82.70			68.52±8.58	68.60±8.20	47.90-85.50		
	ISH	69.21±8.27	72.10±11.50	55.70-80.60			68.17±10.21	68.60±15.50	48.80-90.30		
TBW%	Normal	60.18±5.50	60.94±5.00	41.57-79.47	**H=62.938; p<0.0001**	**R=-0.631; p<0.0001**	60.38±6.92	60.30±10.32	41.08-76.62	**H=97.565; p<0.0001**	**R=-0.552; p<0.0001**
	High-normal	53.93±6.50	54.27±8.55	39.44-65.98			51.65±6.57	52.54±9.62	40.34-69.49		
	HTN	48.65±5.09	48.34±5.20	38.16-60.41			50.52±6.11	50.14±5.81	35.02-62.65		
	ISH	50.69±6.05	52.85±8.32	40.77-59.05			50.68±7.11	50.24±9.84	35.77-66.19		

Data are presented as mean ± standard deviation (M±SD), median ± interquartile range (Me±IQR), and minimum–maximum values. Differences between blood pressure (BP) categories were assessed using the Kruskal–Wallis H test (H), and associations were evaluated using Spearman's rank correlation coefficient (R). Statistically significant results are indicated in bold. Abbreviations: WC – waist circumference; HC – hip circumference; BMI – body mass index; BF% – body fat percentage; MM% – muscle mass percentage; FFM% – fat-free mass percentage; TBW% – total body water percentage; BP – blood pressure; HTN – hypertension; ISH – isolated systolic hypertension.
